# Supplementary figures and images for: Homogenization of Endosymbiont Communities Hosted by Equatorial Corals during the 2016 Mass Bleaching Event
Source: Microorganisms. 2020 Sep 7;8(9):1370. doi: 10.3390/microorganisms8091370 (PMC7564173; doi:10.3390/microorganisms8091370)

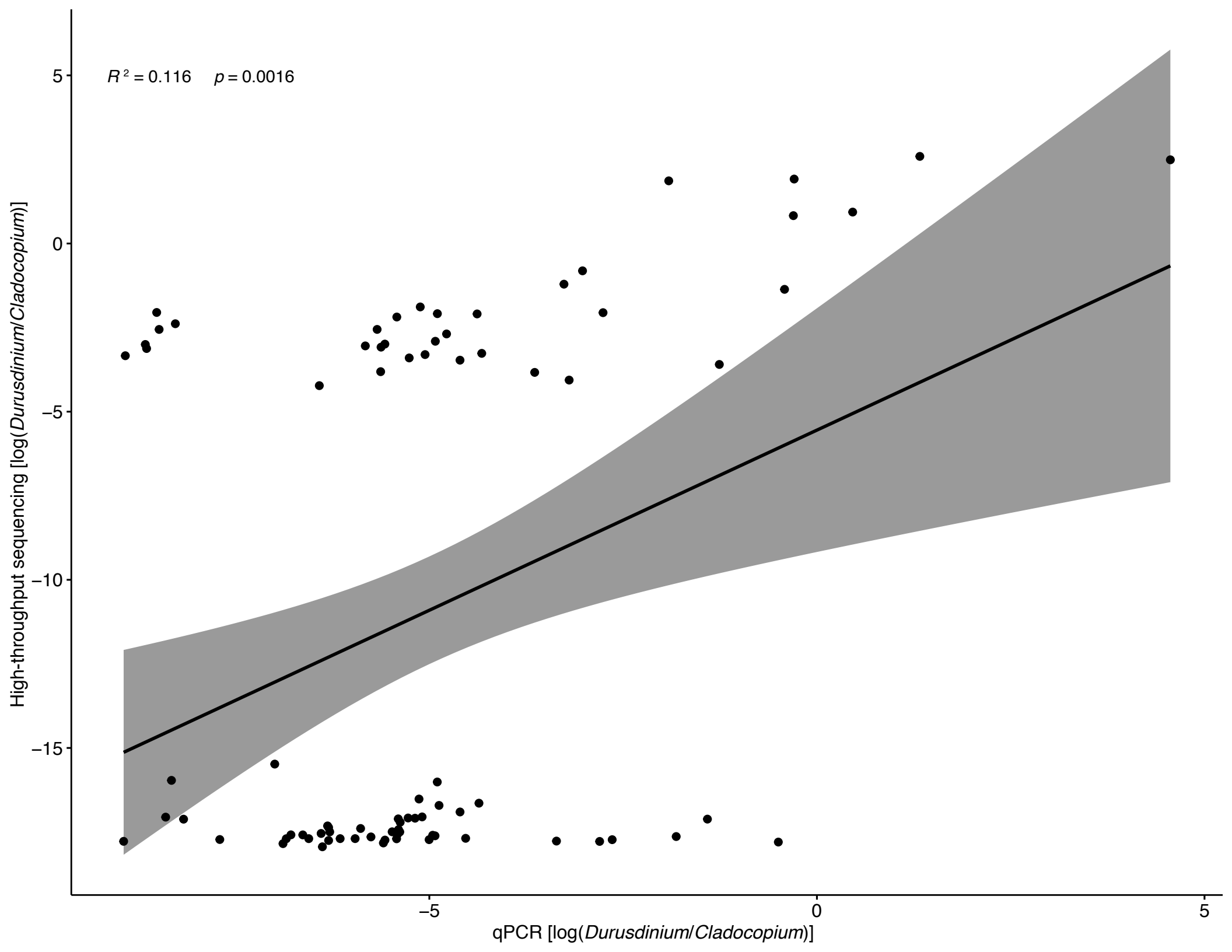

Supplement: Supplementary file 1 [file microorganisms-08-01370-s001.zip › Supplementary Figure 1. Correlation Plot (log values) v3.pdf]
